# Heavy metal driven co-selection of antibiotic resistance in soil and water bodies impacted by agriculture and aquaculture

**DOI:** 10.3389/fmicb.2012.00399

**Published:** 2012-12-14

**Authors:** Claudia Seiler, Thomas U. Berendonk

**Affiliations:** Institute of Hydrobiology, Technische Universität DresdenDresden, Germany

**Keywords:** co-selection, antibiotic resistance, heavy metal, agriculture, aquaculture, farming

## Abstract

The use of antibiotic agents as growth promoters was banned in animal husbandry to prevent the selection and spread of antibiotic resistance. However, in addition to antibiotic agents, heavy metals used in animal farming and aquaculture might promote the spread of antibiotic resistance via co-selection. To investigate which heavy metals are likely to co-select for antibiotic resistance in soil and water, the available data on heavy metal pollution, heavy metal toxicity, heavy metal tolerance, and co-selection mechanisms was reviewed. Additionally, the risk of metal driven co-selection of antibiotic resistance in the environment was assessed based on heavy metal concentrations that potentially induce this co-selection process. Analyses of the data indicate that agricultural and aquacultural practices represent major sources of soil and water contamination with moderately to highly toxic metals such as mercury (Hg), cadmium (Cd), copper (Cu), and zinc (Zn). If those metals reach the environment and accumulate to critical concentrations they can trigger co-selection of antibiotic resistance. Furthermore, co-selection mechanisms for these heavy metals and clinically as well as veterinary relevant antibiotics have been described. Therefore, studies investigating co-selection in environments impacted by agriculture and aquaculture should focus on Hg, Cd, Cu, and Zn as selecting heavy metals. Nevertheless, the respective environmental background has to be taken into account.

## Introduction

The number of infections caused by antibiotic resistant bacteria is rising worldwide (Levy and Marshall, 2004). Because of this critical development associated with the loss of the therapeutic potential of antibiotics, some reports are already describing the start of the post-antibiotic era with the consequence of rising difficulties in the treatment of infectious diseases (Alanis, 2005). The decrease of antibiotic use does not necessarily prevent the spread and maintenance of antibiotic resistance in clinical but also in natural environments (Salyers and Amábile-Cuevas, 1997). Therefore, we need to find additional ways to slow down this alarming development (Aminov, 2010). For this reason, it is essential to fully understand the mechanisms and especially the triggers of the evolution and dissemination of antibiotic resistance.

Since there is evidence for recent exchanges of antibiotic resistance genes between environmental and pathogenic bacteria (Poirel et al., 2002, 2005; Forsberg et al., 2012), research brought the natural environment into focus. It is likely that the spread and evolution of antibiotic resistance is triggered or catalysed by anthropogenic pollutants. It has been proved that antimicrobial agents different from antibiotics have the ability to promote a co-selection process, indirectly selecting for antibiotic resistance (Baker-Austin et al., 2006). Heavy metal contaminations are widely spread, whereby agriculture (Han et al., 2001) as well as aquaculture (Burridge et al., 2010) contributing to that environmental burden.

Additionally, due to agricultural and aquacultural practice antibiotics are transferred to soil and water environments, for example soil being fertilized with antibiotic containing manure and sewage sludge (Heuer et al., 2011). Once the antibiotics reach soil environments they may leach to water environments (Boxall et al., 2002). Although the use of antibiotics added to fish feed in aquacultures decreased significantly after the development of vaccinations (Sørum, 2006), the medication for fish with antibiotics as feed amendments is still in practice. The discharge of heavy metals together with antibiotics from agriculture and animal production-linked ecosystems to the environment may cause a combined effect of selection and co-selection toward antibiotic resistant bacteria. Therefore, soil and water bodies impacted by agriculture and aquaculture are hot spots of the evolution of antibiotic resistant bacteria and require special scientific consideration.

Here, we review the risk for metal driven co-selection of antibiotic resistance by addressing heavy metal sources as well as heavy metal toxicity, with regard to aquaculture and agriculture. Additionally, we will review co-selection mechanisms and identify heavy metal concentrations that potentially induce antibiotic resistance co-selection.

## Farming and aquaculture as sources of heavy metals

The anthropogenic contamination of the environment with heavy metals is a serious problem. Aquaculture (Burridge et al., 2010) and agricultural practices (Han et al., 2002; Nicholson et al., 2003) contribute to this world wide pollution due to diverse applications of metals in feed additives, organic and inorganic fertilizers, pesticides, and anti-fouling products.

Fish farmers frequently use pharmaceuticals (such as antibiotics) and metal containing products to prevent fouling, to feed and to treat fish in order to limit the spread of infections (Burridge et al., 2010). For instance, copper (Cu)-containing materials are applied as anti-fouling agents for farm cages and nets; some cages themselves are made from Cu alloys (Burridge et al., 2010). Therefore, bacterial communities of aquacultures are strongly exposed to the combination of heavy metals and antibiotics. The exposure to both antimicrobial substances may increase the likelihood of selection and co-selection of antibiotic resistance. Moreover, the high nutritional value and the relatively low cost of wastewater, excreta, and sewage sludge convert such heavy metal containing waste to valuable fish feed, especially in developing countries (WHO, 2006). The relevance of heavy metal contaminations in aquaculture has been illustrated by Choi and Cech (1998), who found unexpected high concentrations of mercury (Hg) in fish feed. The enrichment of aquaculture sediments with zinc (Zn) (Morrisey et al., 2000) and Cu (Smith et al., 2005; Burridge et al., 2010) as well as cadmium (Cd) (Dean et al., 2007) and lead (Pb) (Mendiguchía et al., 2006) was reported earlier and is presented in Table 1.

**Table 1:** **Heavy metal concentrations of water, sediment, soil, sewage sludge, and manure**.

**Sample**	**Unit**	**Cd**	**Cr**	**Cu**	**Ni**	**Hg**	**Co**	**Pb**	**Zn**	**Influence**	**References**
**Water**		**Dissolved heavy metal concentration**									
River Hwangryong (KP)	μg L^−1^	0.27	0.38	1.31	0.52	–	0.39	0.21	7.93	Agriculture, rural, urban	Park et al., 2007
Asian rivers	μg L^−1^	–	–	–	0–1.6	–	0–0.5	0–0.8	–	Agriculture, urban	Park et al., 2011
		**Total heavy metal concentration**									
Asian rivers	μg L^−1^	–	–	–	0–5	–	0.3–2.5	0.8–9	–	Agriculture, urban	Park et al., 2011
River Elbe (DE)	μg L^−1^	0.07–0.38	1.2–4.8	4.4–7.4	3.4–4.8	0.04–0.09	–	2.2–5.4	18–35	Agriculture, urban, industry	IKSE, 2006
River Thames (UK)	μg L^−1^	0.03–0.4	–	1.5–30	2.5–15	–	–	2–35	0–70	Urban, agriculture	Power et al., 1999
Loch Craignish (UK)	μg L^−1^	0.7	–	8	–	–	–	–	25	Marine aquaculture	Dean et al., 2007
**Sediment**		**Heavy metal concentration referring to fresh weight**									
River Seine (FR)	mg kg^−1^	2	84	56	–	0.7	–	73	267	Agriculture, urban, industry	Le Cloarec et al., 2011
Chaohu Lake (CN)	mg kg^−1^	0.15–0.50	50–90	15–35	15–40	–	–	10–30	50–150	Agriculture	Tang et al., 2010
		**Heavy metal concentration referring to dry weight**									
San Pedro River (ES)	mg kg^−1^	–	–	15.49	–	–	–	11.32	45.6	Aquaculture	Mendiguchía et al., 2006
Bedfordshire (UK)	mg kg^−1^	–	124.3–180.5	30.2–35	55.5–68.8	–	–	70.5–86.6	–	Agriculture	Quinton and Catt, 2007
River Seine (FR)	mg kg^−1^	–	–	–	–	0.31	–	–	–	Agriculture, urban, industry	Ouddane et al., 2008
River Elbe (DE)	mg kg^−1^	–	–	30–166	12–59	1–6	–	36–185	249–1358	Agriculture, urban, industry	Baborowski et al., 2012
River Medway (UK)	mg kg^−1^	–	–	–	–	0.3	–	–	–	Agriculture, urban, industry	Ouddane et al., 2008
Stewart Island (NZ)	mg kg^−1^	–	–	–	–	–	–	–	665	Marine aquaculture	Morrisey et al., 2000
Loch Craignish (UK)	mg kg^−1^	1.3	–	100	–	–	–	–	450	Marine aquaculture	Dean et al., 2007
**Soil**		**Heavy metal concentration referring to dry weight**									
	mg kg^−1^	0.1–0.4	37.7–59	1–4.3	13.4–17.8	0.1–0.4	–	11.9–21.6	2.7–10.8	Compost	Zhang et al., 2006
	mg kg^−1^	0.2–0.4	33.4–50.2	0.8–2.9	12.7–15	0.1–0.4	–	12.3–16.6	3.5–11.4	Compost	Zhang et al., 2006
	mg kg^−1^	0.1	36	0.6	12.8	0.3	–	11.1	0.7–0.8	NPKS fertilizer	Zhang et al., 2006
	mg kg^−1^	0.2	30.3	0.8	12.1	0.1	–	11.1	2.9	NPKS fertilizer	Zhang et al., 2006
	mg kg^−1^	0.1	35.3	0.6–0.7	13.0	0.2	–	11.0	0.7	PK fertilizer	Zhang et al., 2006
	mg kg^−1^	0.2	30.5	0.6–0.7	12.0	0.1	–	14.1	3.1	PK fertilizer	Zhang et al., 2006
	mg kg^−1^	0.75	–	–	–	–	–	–	–	Sewage sluge	Bergkvist et al., 2003
	mg kg^−1^	–	54.2	130.5	34.2	–	–	–	179.5	Swine compost	Zhao et al., 2006
	mg kg^−1^	–	54.5	108.2	33.2	–	–	–	106.2	N fertilizer	Zhao et al., 2006
	mg kg^−1^	–	56.8	107.0	32.7	–	–	–	103.9	PK fertilizer	Zhao et al., 2006
	mg kg^−1^	0.58–0.62	34.5–35	26.7–27.2	30.8–30.9	–	–	24.1–25.2	66.3–70.7	Biowaste-compost	Erhart et al., 2008
	mg kg^−1^	0.59–0.63	34.7–35.6	25.8–26.8	30.5–32.4	–	–	23.9–24.4	64.8–67.3	NPK-fertilizer	Erhart et al., 2008
	mg kg^−1^	–	58.1–72.5	7.2–9.3	19–21.8	–	–	24.9–28.3	–	Agriculture	Quinton and Catt, 2007
	mg kg^−1^	–	–	149–421	–	–	–	–	–	Cu-pesticide	Scheffer and Schachtschabel, 2010
**Sewage sludge**		**Heavy metal concentration referring to dry weight**									
	mg kg^−1^	1.3–13	–	–	–	–	–	–	–		Bergkvist et al., 2003
	mg kg^−1^	4	97	236	40	–	10	60	1640		Saviozzi et al., 1999
	mg kg^−1^	1.3–2.3	42–71	184–330	30–43	–	–	61–152	1098–1550		Matamoros et al., 2012
	mg kg^−1^	1.74	85.3	223	46.2	2.2	–	83.6	1025		Chen et al., 2012
	mg kg^−1^	10	500	800	80	6	30	500	1700		Manara and Zabaniotou, 2012
	mg kg^−1^	2.83	74.8	190	90.3	–	5.67	21.7	408		Xu et al., 2012
**Limit value agricultural sludge application**		**Heavy metal concentration referring to dry weight**									
	mg kg^−1^	20–40	–	1000–1750	300–400	16–25	–	750–1200	2500–4000		Council Directive 86/278/EEC, 1986
**Manure**		**Heavy metal concentration referring to dry weight**									
Farmyard manure	mg kg^−1^	6	9	66	14	–	4	60	340		Scheffer and Schachtschabel, 2010
Swine compost	mg kg^−1^	–	8.5	221.0	11.6	–	–	–	691.9		Zhao et al., 2006
Biowaste compost	mg kg^−1^	< 0.3–0.7	–	35–90	17–28	–	–	35–101	152–400		Bartl et al., 2002
	mg kg^−1^	< 0.3–0.6	14–40	23–63	13–25	–	–	33–63	145–240		Erhart et al., 2008

–, no data.

Most serious heavy metal contaminations in soils include Cu, Hg, Zn, Pb, and Cd (Han et al., 2002). Land application of metal containing fertilizers, sewage sludge, and liquid manure is common practice in agriculture not only in Europe but also in other regions of the world. Due to those applications heavy metals such as Pb, Hg, Cd, Cu, Zn, chromium (Cr), and nickel (Ni) are transferred to arable soil (Table 1). Because of its bactericidal and fungicidal properties, Cu-containing pesticides are applied in organic and conventional agriculture (Nemecek et al., 2011). Furthermore, metals such as iron (Fe), cobalt (Co), manganese (Mn), Cu, and Zn are applied as nutritional additives in animal feed for livestock farming and fish production in Europe (Commission Regulation 1831/2003/EC, 2003).

A pre-assessment of the environmental impact of Zn and Cu feed amendments in the European Union demonstrated the major role of aquaculture and agriculture as pollution sources of those metals. The applied models predicted that the no effect concentrations of Cu and Zn will be exceeded in some soil and water systems within the next 10–50 years (Monteiro et al., 2010). Moreover, agriculture was identified as the main source of Cu- and Zn-contamination of arable soil in England and Wales (Nicholson et al., 2003). In addition, 30% of the Cd input to the investigated agricultural soil originated from inorganic fertilizers.

## Heavy metal toxicity and resistance

Not all heavy metals are equally toxic to bacteria. Some are important trace metals involved in various physiological functions of the cell. For example Zn, Ni, Cr, Cu, and Co are metals of moderate to high physiological importance. They are essential micronutrients necessary for several cellular functions and components of DNA- and RNA-polymerases (Zn), urease (Ni), cytochrome (Cr) and cytochrom—c—oxidase (Cu). Pb, Cd, Hg, silver (Ag), and gold (Au) have reduced relevance as trace nutrients and they have limited physiological function. Cd and Hg are strong cellular toxins because of their ability to form harmful complexes (Nies, 1999). In contrast, the toxicity of trace metals such as Zn, Ni, Cu, Co, and Cr are strongly dependent on the concentration. As reviewed by Nies (1999), the elements Fe, Mn and molybdenum (Mo) were described as physiologically important with limited toxicity. Metals such as Zn, Ni, Cu, Co, Cr, vanadium (V), and tungsten (W) are toxic elements with metabolic relevance, while the elements Ag, Cd, Hg, Pb, antimony (Sb), and uranium (U) are strong toxins.

The toxicity of heavy metals in the environment strongly depends on the environmental conditions because these conditions influence the valence of the metal ions and therefore their bioavailability. Environmental Cr, for example mainly occurs in two different forms: as Cr^3+^ ion or as the hexavalent Cr associated with oxygen as chromate (for example CrO^2−^_4_). The Cr^3+^ ions are less toxic to bacteria than the chromate (Nies, 1999). Environmental conditions like the pH-value, the concentration of organic matter and the redox potential affect the concentrations and bioavailability of heavy metals in soil, sediment, and water. For instance, the oxygen level influences the redox potential and thereby affects the solubility of some metals. In some water bodies the decomposition of high concentrations of organic matter leads to a reduction of the oxygen level down to anaerobic conditions. Under such conditions the solubility of Cd and Zn is reduced (Schulz-Zunkel and Krueger, 2009). On the other hand, low pH-values increase the solubility of the metals Pb, Cd, and Zn. High contents of organic matter within the sediment act as a sink for some metals: for example Cr and Zn are known to bind to organic matter (Schulz-Zunkel and Krueger, 2009).

In general, the microbial toxicity of heavy metals is due to their chemical affinity to the thiol groups and macrobiomolecules but also depends on the solubility of the metal compound under physiological conditions (Nies, 1999). To avoid cellular damage caused by metal ions, bacteria evolved mechanisms of metal tolerance. There are three general mechanisms which result in heavy metal resistance: the first mechanism is the complex formation or sequestration of toxic metals (Silver and Phung, 1996). Upon metal binding, the concentration of the free toxic ions in the cytoplasm is minimized. Biosorption of toxic metals is known from cell membranes, cell walls and extracellular polymeric substance (EPS) of biofilms (Harrison et al., 2007). For example, the EPS matrix and the contained polysaccharides were reported to bind heavy metals (Teitzel and Parsek, 2003). Thus, the metal tolerance of the bacteria belonging to that biofilm was enhanced. The second mechanism of resistance to toxic metals is the detoxification through reduction of intracellular ions (Nies, 1999). A well understood example is the mercury reductase, encoded by the *merA* gene. This MerA protein reduces Hg^2+^ to the less toxic Hg^0^ (Schiering et al., 1991). Hg^0^ will then diffuse out of the cell, due to its low evaporation point (Nies, 1999). Finally, extrusion of toxic ions by efflux systems is the third mechanism of heavy metal resistance (Nies and Silver, 1995). The cation/proton antiporter Czc, known for example from *Alcaligenes eutrophus*, mediates resistance to the metal ions Cd^2+^, Zn^2+^, and Co^2+^ by extrusion of metals from the cytoplasm though the inner and outer membrane to the surrounding environment (Silver and Phung, 1996). Population wide metal tolerance is increased by persister cells (Harrison et al., 2007). Persister cells mediate time dependent tolerance to toxic metal ions due to upregulation of resistance and stress response genes (Harrison et al., 2007).

Bacterial sensitivity can be quite complex, nevertheless, some generalizations seem to be possible. Gram positive bacteria are described to be more sensitive to toxic metals than gram negative bacteria (Sterritt and Lester, 1980). Moreover, two general microbial toxicity rankings were reported (Nies, 1999; Harrison et al., 2007). In these rankings bacterial susceptibility is described as a function of the particular metal sulfide dissociation constants (pK_*SP*_) (Nies, 1999) and as a function of the standard reduction potentials (ΔE_0_) (Harrison et al., 2007). Those two general toxicity rankings are shown in Table 2. Nevertheless, different types of bacteria show different sensitivities to toxic metals. Even the heavy metal susceptibility of bacteria belonging to the same genera can differ dramatically. As an example, while the growth of one *Aeromonas* isolate is inhibited by a concentration of 100 μg Zn ml^−1^, another strain of the same genera isolated from the same sampling site has the ability to grow up to a concentration of 3200 μg Zn ml^−1^ (Matyar et al., 2010). Further examples showing bacteria and their susceptible to toxic metals are displayed in Table 2. The listed examples did not show the same pattern of toxicity as reported by Nies (1999) and Harrison et al. (2007). This demonstrates that environmental bacteria may adapt to their ecological conditions and may have been selected for certain metal tolerance mechanisms.

**Table 2:** **Toxicity ranking of heavy metals in recent studies**.

**Testorganism**	**Metal sensitivity ranking**	**References**
*Escherichia coli*	Hg^2+^ > Ag^+^/Au^3+^ > CrO^2−^_4_ > Cd^2+^ > Co^2+^/Ni^2+^/Cu^2+^/Zn^2+^ > Pb^2+^/Cr^3+^ > Mn^2+^	Nies, 1999
*Escherichia coli*	Hg^2+^ > Ag^+^ > Cu^2+^ > Cd^2+^ > Zn^2+^ > Ni^2+^ > Mn^2+^	Harrison et al., 2007
*Pseudomonas* spp. and *Aeromonas* spp.	Cd^2+^ > Zn^2+^/Co^2+^/Cu^2+^/Cr^3+^ > Pb^2+^ > Mn^2+^	Akinbowale et al., 2007
*Pseudomonas aeruginosa*	Pb^2+^ > Cu^2+^ > Zn^2+^	Teitzel and Parsek, 2003
*Pseudomonas* spp.	Hg^2+^ > Zn^2+^/Cd^2+^/Ni^2+^/Pb^2+^/Cr^6+^/Cu^2+^/Cr^3+^	Malik and Aleem, 2011
*Escherichia coli*	Cr^6+^ > Cu^2+^/Pb^2+^ > Ni^2+^/Cr^3+^/Co^2+^ > Zn^2+^ > Cd^2+^	Abskharon et al., 2008
*Bacillus* spp.	Hg^2+^ > Cd^2+^ > Zn^2+^	Timoney et al., 1978

Compared are distributions of minimum inhibitory concentrations (MIC) of several metal ions.

## The machinery of co-selection

Since the 1970's, there has been great concern about heavy metals selecting indirectly for antibiotic resistance by co-selection (Koditschek and Guyre, 1974). This indirect selection process is due to a coupling of the resistance mechanisms against antibiotics and heavy metals. Those mechanisms can be coupled physiologically (cross-resistance) and genetically (co-resistance). Cross-resistance describes mechanisms that provide tolerance to more than one antimicrobial agent such as antibiotics and heavy metals (Chapman, 2003). As an example, several multi drug efflux pumps are known to mediate decreased susceptibility toward antibiotics and heavy metals by rapid extrusion of the toxins out of the cell (Martinez et al., 2009). Further well-characterized cross-resistance mechanisms were reviewed by Baker-Austin et al. (2006). Co-resistance is defined as two or more genetically linked resistance genes, meaning that genes responsible for two or more resistances are located next to each other on one mobile genetic element (Chapman, 2003). As an example, Osman et al. (2010) isolated an aquatic bacterium harbouring a plasmid which contained genes conferring resistance to antibiotics and metals like Cr and Co. Due to the close arrangement of the genes it is likely that these genes are subject to a combined transmission in the case of a horizontal gene transfer. A genetic linkage of Cu resistance encoded by the *tcrB* gene, macrolide [*erm*(B)] and glycopeptid resistance (*vanA*) was observed in *Enterococcus faecium* isolated from farm animals (Hasman and Aarestrup, 2002). Here co-resistance to Cu and antibiotics, all applied in farming practice was detected. Macrolides are commonly used in veterinary medicine (Grave et al., 2010) and glycopeptide antibiotics have been used as growth promoters for animal production in the past. Nowadays glycopeptide antibiotics, such as vancomycin, belong to the group of last resort antibiotics in human medicine. Thus, this genetic linkage found by Hasman and Aarestrup (2002) could be an example for a Cu-induced spread of resistance to antibiotics relevant in veterinary and human medicine. Furthermore, *Aeromonas salmonicida* subsp. *salmonicida* isolated from Atlantic salmon (*Salmo salar*) from aquaculture facilities was identified carrying Hg (*mer* operon) and multiple antibiotic resistance genes (*aadA7, sulI, sulII, floR, tetA, tetR, strA, strB*, and *bla*_*CMY*−2_) on an IncA/C plasmid (McIntosh et al., 2008). This was the first finding of plasmid associated resistance to florfenicol (*floR*), an antibiotic usually used to treat furunculosis in aquacultures.

Integrons are genetic elements capable of acquiring and exchanging DNA fragments named gene cassettes. Furthermore, class 1 integrons are assumed to catalyse co-selection because they frequently contain gene cassettes that mediate resistance to antibiotics and they are frequently found in contaminated habitats. Presumably these integrons mediate a selective advantage to bacteria that occur under stressful environmental conditions, for example due to toxic metals. This assumption is supported by several studies which discovered elevated abundance of class 1 integrons in aquatic environments contaminated with heavy metals (Wright et al., 2008; Rosewarne et al., 2010). Further indications for a co-resistance mechanism in fresh water bacteria were given by Gillings et al. (2008) and Stokes et al. (2006). Both publications document class 1 integrons which are closely located to genes coding for the multi drug efflux pump czcA. This efflux pump is known to extrude the metal ions Zn^2+^, Cd^2+^, and Co^2+^.

Moreover, environmental pollution by heavy metals not only triggers co-selection processes, but also increases the level of tolerance to antibiotics due to co-regulation of resistance genes. Heavy metal ions are known to co-regulate genes responsible for antibiotic resistance and decrease antibiotic susceptibility (Baker-Austin et al., 2006). For example, the soxS protein is a regulator for the AcrAB efflux system in *Escherichia coli*. Under oxidative stress, for instance caused by several metal ions like Cr_2_O^−^_7_ and Cu^2+^, *soxS* is upregulated (Harrison et al., 2009). The subsequently increased production of the AcrAB efflux system additionally mediates enhanced tolerance toward antibiotics such as chloramphenicol, tetracyclin, novobiocin, nafcillin, and oxacillin.

Studies investigating co-selection in the environment frequently show the correlation of increased heavy metal concentrations with increased phenotypic or genotypic antibiotic resistance (Table 3). However, some studies indicate that increasing heavy metal concentrations lead to a decrease of antibiotic resistance (Stepanauskas et al., 2005; Tuckfield and McArthur, 2008; Hölzel et al., 2012). These contradicting results were investigated by Hölzel et al. (2012). In consequence of the addition of mercury chloride (HgCl_2_) to the antimicrobial test procedure the minimum inhibitory concentration (MIC) for a wide range of antibiotics decreased. This observation could be due to an interaction of Hg with enzymes or nucleic acids which cause antibiotic resistance. HgCl_2_ could also have a co-toxic effect with antibiotics that interfere with ribosomes because the regeneration of the Hg-degraded enzyme would be inhibited. Furthermore, Hölzel et al. (2012) mentioned also a possible metal induced shift within the bacterial community toward Hg tolerant bacteria whereby the benefit of antibiotic resistance in the presence of antibiotics would be outcompeted. The increased antibiotic susceptibility in consequence of Hg exposure could also play a role in the observations of other field studies. For example, Tuckfield and McArthur (2008) observed decreasing microbial aminoglycoside resistance at sites with increased Hg concentrations.

**Table 3:** **Heavy metal concentrations of studies investigating co-selection in laboratory and field experiments**.

**Sample**	**Study type**	**Unit**	**Cd**	**Cr**	**Cu**	**Ni**	**Hg**	**Co**	**Pb**	**Zn**	**References**
**Water**			**Dissolved heavy metal concentration**								
	Field	μg L^−1^	0.03–0.13	–	1.5–2.5	0.6–2.7	–	–	0.02–0.06^*a*^	18.7–26^*a*^	Wright et al., 2006
	Field	μg L^−1^	0.08–1.12	–	1.27–12.71	0.29–11.74	–	0.05–3.54	0.15–0.2	19.61–98.07	Stepanauskas et al., 2005
	MCC_waterDC_	μg L^−1^	0.03	–	1.5	0.29	–	0.05	0.15	19.61	
			**Total heavy metal concentration**								
	Laboratory	mg L^−1^	0–0.11	–	–	5.9–58.7	–	–	–	–	Stepanauskas et al., 2006
	MCC_waterTC_	mg L^−1^	0.11	–	–	58.7	–	–	–	–	
**Sediment**			**Heavy metal concentration referring to fresh weight**								
	Field	mg kg^−1^	0.2–1.6^*a*^	58.4–197^*a*^	11.6–869	–	–	3.3–15.6	17.4–108	46.1–800	Graham et al., 2010
	MCC_sedFW_	mg kg^−1^	–	–	11.6	–	–	3.3	17.4	46.1	
			**Heavy metal concentration referring to dry weight**								
	Field	mg kg^−1^	–	–	–	–	0.01–0.09	–	–	–	McArthur and Tuckfield, 2000
	Field	mg kg^−1^	1–2.5	–	11.5–50	–	0.8–1.5	–	–	42.5–135.8	Timoney et al., 1978
	MCC_sedDW_	mg kg^−1^	1	–	11.5	–	0.01	–	–	42.5	
**Soil**			**Heavy metal concentration referring to fresh weight**								
	Field	mg kg^−1^	–	–	116.7	–	–	–	–	–	Berg et al., 2005
	MCC_soilFW_	mg kg^−1^	–	–	116.7	–	–	–	–	–	
			**Heavy metal concentration referring to dry weight**								
	Field	mg kg^−1^	–	–	3172	–	–	–	–	–	Berg et al., 2010
	Field	mg kg^−1^	–	0–250	0–140	0–100	–	0–140^*a*^	10–1000^*a*^	0–38^*a*^	Knapp et al., 2011
**Manure**			**Heavy metal concentration referring to dry weight**								
	Field	mg kg^−1^	5.3^*a*^	32.0^*a*^	11.79	97.3^*a*^	0.01^*a*^	–	2^*a*^	22.75	Hölzel et al., 2012
	MCC_manureDW_	mg kg^−1^	–	–	11.79	–	–	–	–	22.75	

–, no data.

a metal concentration without correlation or in negative correlation to antibiotic resistance.

MCC_waterDC_, minimum co-selective concentration referring to dissolved metals in water.

MCC_waterTC_, minimum co-selective concentration referring to total metals in water.

MCC_sedFW_, minimum co-selective concentration referring to fresh weight of sediment.

MCC_sedDW_, minimum co-selective concentration referring to dry weight of sediment.

MCC_soilFW_, minimum co-selective concentration referring to fresh weight of soil.

MCC_manureDW_, minimum co-selective concentration referring to dry weight of manure.

## Risk assessment for metal driven co-selection

To assess the risk for the co-selection of antibiotic and heavy metal resistance, two datasets of heavy metal concentrations were compared. One of the datasets is shown in Table 3, containing metal concentrations observed in studies that investigated co-selection in laboratory and field experiments. The second dataset is shown in Table 1, containing heavy metal concentrations that were measured in various environmental compartments which are impacted by agriculture and/or aquaculture. Additionally, we adapted a concept of the MIC originating from the antimicrobial susceptibility testing in clinical settings. The MIC is defined as the antibiotic concentration that is needed to inhibit bacterial growth. If this MIC has been increased above an epidemiological cut-off value of a bacterial strain the strain will be defined as antibiotic (microbiological) resistant (URL: http://www.eucast.org). In this context the minimum heavy metal concentration which correlates with a detection of increased bacterial antibiotic resistance, was specified as the minimum co-selective concentration (MCC) of a metal (Table 3). Additionally, specific MCCs to every environmental compartment and the respective analytic detection method of the metals (for example for dissolved and total metal concentrations or metal content referring to dry or fresh weight of the solid samples) were defined (Table 3). Moreover, the MCCs for each metal were subsequently compared to the heavy metal levels found within the corresponding environmental compartments (Table 4). Environmental metal concentrations that exceeded the corresponding MCC were considered as potential drivers of co-selection of antibiotic resistance in the environment.

**Table 4:** **Summary of all studies for which the MCCs were applied**.

		**Ratio (heavy metal concentration ≥ MCC/heavy metal concentration < MCC)**							
**Sample**	**Applied MCC**	**Cd**	**Cr**	**Cu**	**Ni**	**Hg**	**Co**	**Pb**	**Zn**
**Water**	MCC_waterDC_	1/0	–	0/1	2/0	–	2/0	2/0	–
	MCC_waterTC_	0/3	–	–	0/3	–	–	–	–
**Sediment**	MCC_sedFW_	–	–	2/0	–	–	–	–	–
	MCC_sedDW_	1/0	–	4/0	–	3/0	–	–	4/0
**Manure**	MCC_manureDW_	–	–	4/0	–	–	–	–	4/0
**Sewage sludge**	MCC_manureDW_	–	–	5/0	–	–	–	–	5/0

–, no data.

MCC_waterDC_, minimum co-selective concentration referring to dissolved metals in water.

MCC_waterTC_, minimum co-selective concentration referring to total metals in water.

MCC_sedFW_, minimum co-selective concentration referring to fresh weight of sediment.

MCC_soilFW_, minimum co-selective concentration referring to fresh weight of soil.

MCC_soilDW_, minimum co-selective concentration referring to dry weight of soil.

MCC_manureDW_, minimum co-selective concentration referring to dry weight of manure.

Illustrated are ratios, which show the number of studies where the heavy metal concentrations were above the MCC versus the number of studies where the heavy metal concentrations were below the MCC.

In the natural water environment (water and sediment) Cd, Cu, Ni, Hg, Co, Pb, and Zn frequently reach levels that exceed their respective MCC values (Table 4) and therefore, may drive co-selection. While there are several studies available investigating co-selection in the water environment, there are only a few publications considering soil environments (Berg et al., 2005, 2010; Knapp et al., 2011). Thus, the assessment of the risk for the co-selection of antibiotic and heavy metal resistance in soil is limited to Cr, Cu, and Ni (Table 3). Furthermore, the data of Knapp et al. (2011) does not allow extracting MCCs of metals because the lowest metal concentrations that may caused the increase in antibiotic resistance gene abundance are not shown. However, these results provide evidence for Cr, Cu, and Ni driven co-selection of antibiotic resistance in soil. Some Cu and Cr levels of the reviewed arable soil samples (Table 1) are similar or even higher than the levels observed by Knapp et al. (2011) (Table 3). Sewage sludge and manure are part of this risk assessment because those organic fertilizers themselves could facilitate metal driven co-selection of antibiotic resistance before entering soil environments and they might additionally transfer metals to arable soil. Cu and Zn concentration of sewage sludge and manure frequently exceeded the MCCs of manure (Table 4). Moreover, the limit values for heavy metal concentrations of sewage sludge for the use in agriculture (Council Directive 86/278/EEC, 1986) are much higher than the MCCs of manure (Tables 1 and 3). As mentioned earlier in this article, the use of Zn and Cu in animal farming and agriculture is common and those metals have been investigated in all considered environmental compartments. The concentrations for both metals exceed their MCCs for some water, sediment, sewage sludge, and manure samples. In soil Cu levels reach concentrations that are reported as potentially co-selective for antibiotic resistance genes (Knapp et al., 2011). In contrast, a Zn MCC for soil samples could not be evaluated because Knapp et al. (2011) did not detect increasing abundance of antibiotic resistance genes in correlation with elevated Zn concentrations. However, the Zn concentrations of soil samples investigated by Knapp et al. (2011) were relatively low compared to other soils (Table 1) and maybe within the no effect range. In summary, all considered heavy metals (frequently Cu and Zn) reach concentrations above their MCCs in the different environmental compartments. Therefore, the analysis of the data suggests that heavy metal concentrations in soil and water bodies occasionally reach levels that might drive a co-selection of antibiotic resistance.

This risk assessment of heavy metal driven co-selection is based on MCCs which are derived from positive correlations of increased metal concentrations with increased antibiotic resistance. This risk assessment provides a tool to estimate at which levels environmental metal concentrations may cause the dissemination of microbial antibiotic resistance due to co-selection. Ideally such a risk assessment would be conducted under laboratory conditions, as it is currently the case for the determination of the MIC. For the purpose of this study this was not possible as we wanted to review existing studies and included laboratory and field data, in order to detect a first pattern or synthesis on heavy metal induced co-selection of antibiotic resistance in the field. The results of the MCC analysis for such data need to be carefully interpreted, mainly because positive correlations between metal levels and antibiotic resistance could also be false positive. This would be the case, if another selection pressure (for example by antibiotics) would be the trigger of the observed selection and not the co-occurring metal.

In order to better assess the risk of co-selection of antibiotic and heavy metal resistance more research is necessary. Only a limited number of studies is available that investigated the co-selection in water and soil environments and additionally measured heavy metal concentrations (Table 3). Especially for soil environments, there is only one multi metal study on co-selection and metal contamination (Knapp et al., 2011). The knowledge about the natural background of antibiotic resistance gene abundance (resistome) in the different environments is also limited. Thus, we cannot distinguish between the natural resistome and an elevated abundance of antibiotic resistance genes in different environmental samples. Therefore, it is difficult to detect an increase of antibiotic resistance genes in field studies. Further research should relate to the MCCs of heavy metals. Although we were able to derive the MCC values form recent studies, further research in field and laboratory experiments is urgently requested to broaden the database of co-selective concentrations. Stepanauskas et al. (2006) investigated such a selective concentration of Ni and Cd in a lab experiment. Nevertheless, the metal concentrations observed as minimum selective concentrations in those microcosms were much higher compared to the Ni and Cd levels observed in environmental water samples (Tables 1 and 3). This difference requires further investigation and may be an artifact of the growth in the laboratory since laboratory grown bacteria usually have better conditions than their environmental counterparts.

While our MCC approach provides a first step toward a unifying concept for analyzing co-selection of antibiotic resistance through heavy metals, there is an urgent need to extend this approach to a comprehensive risk assessment. The need of such a risk assessment is illustrated in our results, which show that in all considered environmental compartments (water, sediment, and soil) as well as sewage sludge and manure, one or more heavy metals reach concentrations that may lead to a metal driven co-selection of antibiotic resistance.

## Conclusion

Concluding all these facts concerning the heavy metal driven co-selection of antibiotic resistance, metals such as Cd, Hg, Cu, and Zn are of great importance in water and soil environments that are influenced by agriculture and aquaculture. These metals are moderately to highly toxic to bacteria; they reach the environment and they accumulate to selective concentrations. Additionally, they can trigger co-selection of antibiotic resistance because responsible co-selection mechanisms that mediate resistance to these heavy metals and clinically as well as veterinary relevant antibiotics have already been described. Therefore, the elimination of antibiotics from the list of animal feed additives as growth promoters was a step in the right direction. Further steps need to be taken to reduce the alarming spread of antibiotic resistance genes. In addition to the avoidance of antibiotics in livestock farming and aquaculture, further antimicrobial agents such as heavy metals should be considered. These metals have the potential to act as a selective pressure that forces the proliferation and evolution of antibiotic and heavy metal resistance in the natural environment. With the exception of the above mentioned studies, investigations which explicitly test for the co-selection of heavy metals and antibiotics used in animal farms and aquaculture are still scarce. Future studies investigating heavy metal driven co-selection of antibiotic resistance in soil and water bodies impacted by agriculture and aquaculture should focus on Hg, Cd, Cu, and Zn as co-selecting factors for the evolution of antibiotic resistances. Nevertheless, the respective environmental background has to be taken into account.

### Conflict of interest statement

The authors declare that the research was conducted in the absence of any commercial or financial relationships that could be construed as a potential conflict of interest.
